# A rare case report of pancreatic low-grade fibromyxoid sarcoma (LGFMS)

**DOI:** 10.1186/s40792-023-01662-3

**Published:** 2023-07-03

**Authors:** Eric J. Weiler, Thomas Murickan, Crystal N. Drayer, Sajjaad H. Samat, Michael A. Kia

**Affiliations:** 1 Sparrow Hospital, 1200 E, Michigan Ave, #255, Lansing, MI 48912 USA; 2 University of Nebraska Medical Center, 983285 Nebraska Medical Center, Omaha, NE 68198 USA; 3 UnityPoint Clinic General Surgery, 2730 Pierce St. Suite 402, Sioux City, IA 51104 USA; 4 McLaren Hospital, 3500 Calkins Rd, Flint, MI 48532 USA

**Keywords:** Pancreas, Pancreatic cancer, Sarcoma, Low-grade fibromyxoid sarcoma, LGFMS

## Abstract

**Background:**

Low-grade fibromyxoid sarcoma (LGFMS) is an uncommon neoplasm generally affecting muscle tissue. It presents rarely in abdominal viscera and even more rarely occurs in the pancreas. All types of pancreatic sarcomas are uncommon, and LGFMS is a rarer still. We present the case of an LGFMS in the pancreas. Because of its rarity, there are no guidelines for appropriate treatment or summations of the natural course of this illness.

**Case presentation:**

We present the case of a 49-year-old female who presented with epigastric pain. She had a prior history of three episodes of acute pancreatitis many years earlier. A CT revealed a pancreatic body mass, which was biopsied. Pathology returned LGFMS. The patient underwent a distal pancreatectomy and splenectomy. She did well after the case and did not require further intervention.

**Conclusion:**

Though it is exceedingly rare, cases of pancreatic LGFMS should be reported in order to guide clinical decisions. LGFMS has been shown to have high malignant potential in other tissues, and there is no reason to think pancreatic masses will be different. By building a body of evidence about these rare tumors, patient care will benefit.

## Background

To date, there are only three cases of pancreatic low-grade fibromyxoid sarcoma (LGFMS) reported in literature. Primary pancreatic tumors are most often epithelial in origin, with adenocarcinoma accounting for roughly 90 percent of tumors. Pancreatic neuroendocrine tumors (PNETs) only account for three percent. Pancreatic sarcomas account for less than 0.1% of pancreatic malignancies and tend to affect individuals in their 4th or 5th decades. Patients present with symptoms similar to other pancreatic masses, such as abdominal pain, nausea, vomiting or painless jaundice [1]. Sarcomas in general tend to be aggressive with a poor prognosis. One study of primary pancreatic leiomyosarcoma found a five-year mortality rate of 77.8 percent [2]. The literature describes many types of mesenchymal pancreatic tumors other than LGFMS, including leiomyosarcoma, undifferentiated pleomorphic sarcoma, carcinosarcoma, and clear cell sarcoma [1–4].

Low-grade fibromyxoid sarcoma (LGFMS) is a rare tumor generally arising from soft tissues of the inguinal region, thoracic wall, and shoulder [5]. LGFMS has also been described in the mesentery of the small bowel, mesocolon, colon, and liver [6–9]. Similar to other sarcomas, it often affects adults in the fourth decade. Even when occurring in muscle tissue, it is rare, with around 150 cases reported since it was first described in 1987 [5]. Microscopically, it appears as a bland spindle cell neoplasm. [10]. The cells are in a fascicular or whorled pattern, with low mitotic figures and rare nuclear pleomorphism [11]. It is immunoreactive for glycoprotein MUC4 [9, 10]. LGFMS has previously been noted to lack distinctive features on computed tomography (CT) scan. On CT it is generally isoattenuating to muscle and heterogeneously enhancing [11]. While its pathology appears benign, LGFMS has a predilection for local recurrence and distant metastasis, at least when described in muscle tissue. Treatment is generally wide local excision, which often must be repeated. It is resistant to conventional chemotherapy and radiation [10].

## Case presentation

A 49-year-old female with prior medical history of gastroesophageal reflux disease and 20-pack-year smoking presented to the emergency room with complaints of vague abdominal pain for approximately one month prior. She described the pain as dull to sharp, concentrated in the epigastrium with radiation to the back. The patient had no other complaints. She denied nausea, vomiting, anorexia, weight loss, and changes in bowel function. She also had a history of three episodes of acute pancreatitis. The first was 21 years prior to presenting at the ER and was due to gallstones. A cholecystectomy was performed shortly thereafter. She had two more episodes of acute pancreatitis thereafter, the most recent 13 years prior to presenting to the ER. She denied past or present alcohol use. Imaging from these past admissions was unavailable.

**Fig. 1:**
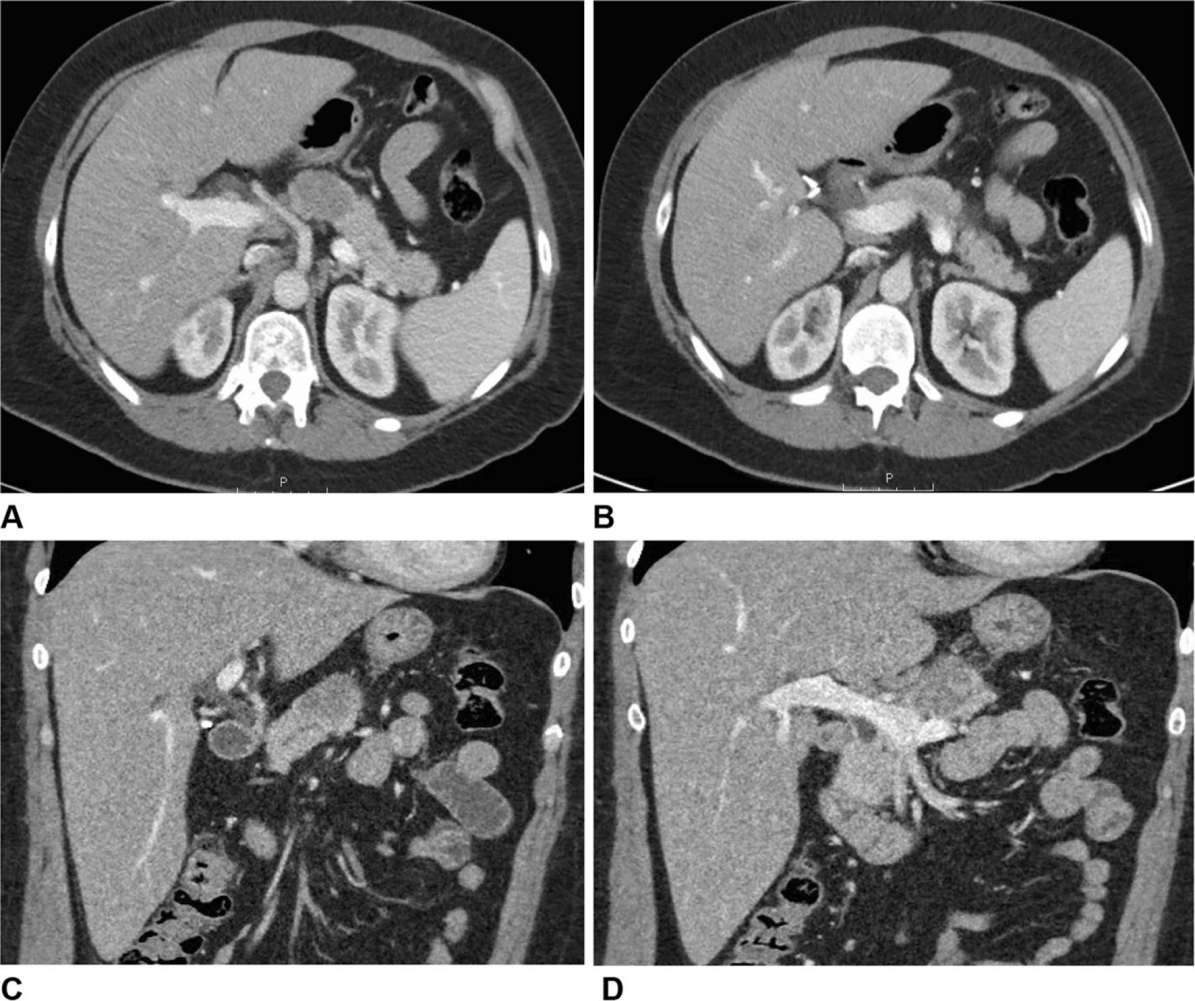
CT scan abdomen/pelvis with and without contrast. Axial (**A**, **B**) and coronal (**C**, **D**) views demonstrating pancreatic mass

A CT at the time of presentation to the emergency room revealed a hypodense pancreatic body mass (Fig. 1). An esophagogastroduodenoscopy (EGD) with endoscopic ultrasound (EUS) was performed, revealing distal gastritis as well as a 3.4 × 2.6 cm pancreatic body mass. There were multiple subcentimeter lymph nodes in the porta hepatis and the gastrohepatic ligament, but no celiac lymphadenopathy. Biopsy of the mass revealed a low-grade spindle cell neoplasm consistent with low-grade fibroid myxoid sarcoma. The patient was discharged with plans for elective surgery in 10 days. She presented to the ER one day before her scheduled surgery complaining of worsening abdominal pain. On presentation, the patient’s labs—including WBC, Hgb, lipase, bilirubin, LFTs, and tumor markers were all within normal limits.

She was admitted and an open distal pancreatectomy with splenectomy was performed without complications. Due to the rarity of the tumor and malignant potential of pancreatic sarcomas generally, a spleen-preserving procedure was decided against. Six peripancreatic lymph nodes were taken with the specimen, with no further lymph node dissection. Frozen section revealed negative margins. The patient had an uncomplicated postoperative course and was discharged home on postoperative day 5. The patient received post-splenectomy vaccines before discharge, which included haemophilus b conjugate, pneumococcal 23 polyvalent, and meningococcus ACWY.

**Fig. 2:**
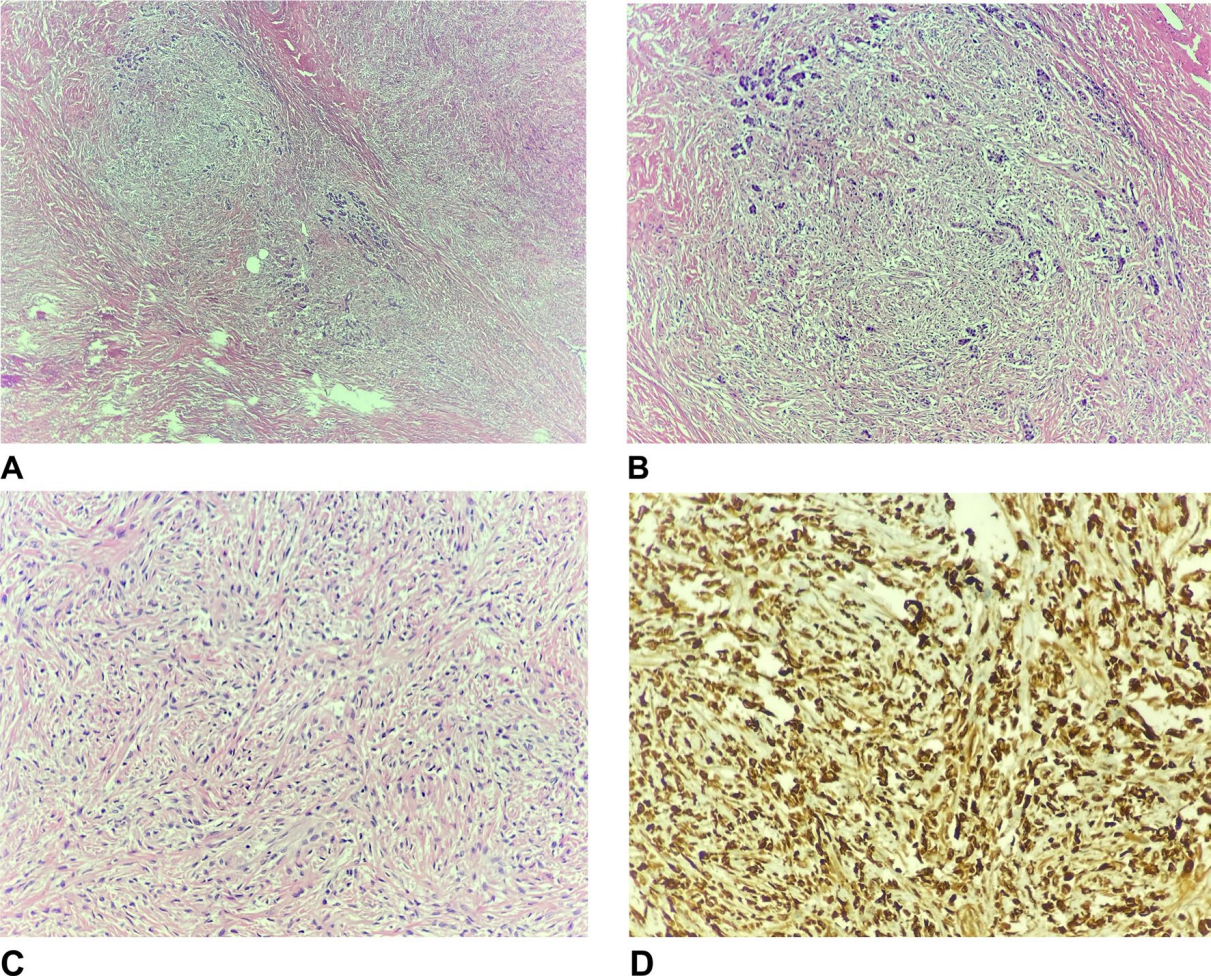
Pathology slides. **A** 2 × low grade view. Spindle cell proliferation expanding pancreatic acini with abrupt transition from collagenized stroma to myxoid areas. **B** 4x. A pancreatic acinar lobule is expanded by spindle cell proliferation. **C** 10x. Spindle cells proliferation with mild cytological atypia in storiform growth pattern. **D** 10x. MUC-4 immunostain is diffusely and strongly positive

On final pathology, the pancreatic mass was measured to be 2.7 × 3.4 × 3.2 cm and identified as a low-grade spindle cell lesion with a sclerotic background, consistent with fibromyxoid sarcoma (Fig. 2). The specimen was sent to a ertiary center, where the diagnosis of fibromyxoid sarcoma with sclerotic and myxoid components was confirmed. The presence of MUC4 marker was identified while the specimen was negative for CD34, STAT6, and S100 protein. This further confirmed presence of a low-grade fibromyxoid sarcoma. The specimen showed negative margins and six benign lymph nodes. The spleen was unremarkable. A portion of omentum sent with the specimen was also negative for metastases.

The patient returned to the office for a two-week follow-up visit and was doing well with no complaints. It was determined that chemotherapy and radiation were not indicated. She was seen at a 4-week follow-up visit complaining of abdominal pain. A CT was done and showed expected postoperative changes, negative for any acute process. At two months post-op, the patient was seen and was doing well. No further follow-up was arranged.

## Discussion

Pancreatic LGFMS rarely appears in the literature. Our review revealed only three cases of primary LGFMS of the pancreas (Table 1). One case report identified a LGFMS with sclerosing features in a 58-year-old man with a history of prostate cancer and radiation. A CT for kidney stones incidentally revealed a six cm mass in the head of the pancreas and he underwent a Whipple procedure. The histology of the mass showed features of LGFMS and sclerosing epithelioid fibrosarcoma [11]. Another report described a 53-year-old patient with a seven cm pancreatic body mass who underwent distal pancreatectomy and external beam radiation. There was invasion of the splenic vein found in the specimen but not lymphatics. He was recurrence free at the time of publishing, but the timeline is unclear [12]. Another paper described LGFMS in the head of the pancreas of a 56-year-old woman who eventually died of complications six months following a Whipple procedure [13]. We found no further examples of pancreatic LGFMS in the literature. Nevertheless, LGFMS should be part of the differential diagnosis for any pancreatic mass of uncertain type if the workup does not reveal the pathology. A notable feature of this case is our patient had several remote episodes of acute pancreatitis. It is unclear if there is an association between LGFMS of the pancreas and acute pancreatitis.

**Table 1:** Summary of literature review and notable features of cases

Case report	Age	Sex	Location	Treatment	Long-term outcome
Kramer et al. (2020)	58	M	Head	Whipple procedure	Unknown
Collins et al. (2014)	53	M	Body	Distal pancreatectomy Radiotherapy	Unknown
Colovic et al. (2008)	56	F	Head	Whipple procedure	Death from complications six months post-op

Because of its rarity, the guidelines for treatment are not clear. There is presently no standard of care for pancreatic LGFMS as there are so few cases. It is unclear if chemotherapy or radiotherapy are useful adjuncts. Drawing on experience from other types of sarcomas, it is possible that LGFMS has strong malignant potential. Given the propensity of LGFMS to recur, we might have considered surveillance imaging for our patient. But given the lack of guidelines, it was difficult to determine if further surveillance or adjuvant treatment was needed, especially considering we obtained clear margins and no evidence of lymphatic or vascular invasion.

## Conclusion

Our case involved a 49-year-old female suffering epigastric pain from a LGFMS tumor of the pancreatic body, successfully treated with a distal pancreatectomy. It was possibly associated with her prior episodes of acute pancreatitis, although this is uncertain given the paucity of literature on this subject. Cases of LGFMS should be reported in the future to help determine the features, natural course, and best methods of treatment for these rare and potentially malignant tumors.

## Data Availability

Not applicable as this is a case report; all data presented were extracted from the patient’s hospital chart.

## Abbreviations

CT: Computed tomography
LGFMS: Low-grade fibromyxoid sarcoma
EGD: Esophagogastroduodenoscopy
EUS: Endoscopic ultrasound
PNET: Pancreatic neuroendocrine tumor
